# Prenatal and Early Childhood Exposure to Tetrachloroethylene and Adult Vision

**DOI:** 10.1289/ehp.1103996

**Published:** 2012-07-11

**Authors:** Kelly D. Getz, Patricia A. Janulewicz, Susannah Rowe, Janice M. Weinberg, Michael R. Winter, Brett R. Martin, Veronica M. Vieira, Roberta F. White, Ann Aschengrau

**Affiliations:** 1 Department of Epidemiology, Boston University School of Public Health, Boston, Massachusetts, USA; 2 Department of Ophthalmology, Boston University School of Medicine, Boston, Massachusetts, USA; 3 Department of Biostatistics; 4 Data Coordinating Center, and; 5 Department of Environmental Health, Boston University School of Public Health, Boston, Massachusetts, USA; 6 Department of Neurology, Boston University School of Medicine, Boston, Massachusetts, USA

**Keywords:** color vision, contrast sensitivity, perchloroethylene, tetrachloroethylene

## Abstract

Background: Tetrachloroethylene (PCE; or perchloroethylene) has been implicated in visual impairments among adults with occupational and environmental exposures as well as children born to women with occupational exposure during pregnancy.

Objectives: Using a population-based retrospective cohort study, we examined the association between prenatal and early childhood exposure to PCE-contaminated drinking water on Cape Cod, Massachusetts, and deficits in adult color vision and contrast sensitivity.

Methods: We estimated the amount of PCE that was delivered to the family residence from participants’ gestation through 5 years of age. We administered to this now adult study population vision tests to assess acuity, contrast sensitivity, and color discrimination.

Results: Participants exposed to higher PCE levels exhibited lower contrast sensitivity at intermediate and high spatial frequencies compared with unexposed participants, although the differences were generally not statistically significant. Exposed participants also exhibited poorer color discrimination than unexposed participants. The difference in mean color confusion indices (CCI) was statistically significant for the Farnsworth test but not Lanthony’s D-15d test [Farnsworth CCI mean difference = 0.05, 95% confidence interval (CI): 0.003, 0.10; Lanthony CCI mean difference = 0.07, 95% CI: –0.02, 0.15].

Conclusions: Prenatal and early childhood exposure to PCE-contaminated drinking water may be associated with long-term subclinical visual dysfunction in adulthood, particularly with respect to color discrimination. Further investigation of this association in similarly exposed populations is necessary.

Tetrachloroethylene (PCE; perchloroethylene) is a synthetic chemical used in dry cleaning solutions, adhesives, and metal degreasers [U.S. Environmental Protection Agency (EPA) 2008]. High levels of PCE have been extracted from conventionally dry-cleaned fabrics, making such fabrics a common source of exposure to the general population (Sherlach et al. 2011). Improper disposal following industrial use has also made PCE a frequent ground- and surface-water contaminant (U.S. EPA 2008), further contributing to low-level exposure among the general population (Brugnone et al. 1994).

Adverse neurological effects among adults following exposure to low PCE levels are well documented and include decreases in attention, cognitive function, and memory (Altmann et al. 1995; Echeverria et al. 1995; Ferroni et al. 1992; Schreiber et al. 2002; Seeber 1989). Subclinical visual impairments may be sensitive indicators of insults to the central nervous system from exposure to neurotoxicants (Gobba 2000). Decrements in contrast sensitivity have been observed in workers exposed to mixed solvents and in residents of buildings with PCE dry cleaners (Broadwell et al. 1995; Donoghue et al. 1995; Frenette et al. 1991; Hudnell et al. 1996; Schreiber et al. 2002), as have decrements in color discrimination (Campagna et al. 1995, 1996; Castillo et al. 2001; Cavalleri et al. 2000; Fallas et al. 1992; Gobba 2000; Gobba et al. 1991; Mergler et al. 1991; Raitta et al. 1978; Zavalic et al. 1998a, 1998b, 1998c).

The impacts of gestational and early childhood solvent exposure on vision are not as well studied. One study found an increased incidence of impaired color discrimination following gestational exposure to organic solvents (Till et al. 2001). Similar visual disturbances have also been observed following prenatal exposure to neurotoxic agents, such as methylmercury (Grandjean et al. 2001), cocaine (Block et al. 1997), and alcohol (Hug et al. 2000; Stromland and Hellstrom 1996).

The objective of the present study was to assess visual functioning—specifically visual acuity, contrast sensitivity, and color discrimination—in adults exposed during gestation and early childhood to PCE-contaminated drinking water.

## Methods

*Selection of study population, follow-up, and enrollment*. The study population was selected from individuals enrolled in a cohort study on the effects of early-life exposure to PCE-contaminated drinking water. Participants provided written consent to undergo the vision examinations. This study was approved by the Institutional Review Boards of the Massachusetts Department of Public Health and Boston University Medical Center, and by the 24A/B/11B Review Committee at the Department of Public Health.

Participants were born from 1969 through 1983 (inclusive) to parents residing in one of eight towns in the Cape Cod region of Massachusetts (Barnstable, Bourne, Brewster, Chatham, Falmouth, Mashpee, Provincetown, and Sandwich). These towns had a proportion of their drinking-water distribution systems outfitted with asbestos-cement (AC) pipes whose vinyl liner (VL) was improperly cured and so it leached PCE into the water supply. These pipes were installed from 1968 through early 1980, according to the town’s replacement and expansion needs. Approximately 660 miles of VL/AC pipes were installed in Massachusetts, a large portion on Cape Cod. The irregular installation pattern led to a wide range of PCE levels in the drinking water; measurements taken in 1980 ranged from 1.5 µg/L to 7,750 µg/L (Demond 1982). Reported levels of other water contaminants were generally low during this period. Because replacing the VL/AC pipes was prohibitively expensive, officials initiated a program of flushing and bleeding to reduce PCE levels to below 40 µg/L, the suggested action level for remediation when the contamination was discovered in 1980.

Selection and enrollment of the cohort have been described previously (Aschengrau et al. 2008). “Exposed” individuals were identified as births to women who lived in affected homes by cross-matching the address on their birth certificates to geographic information systems data on VL/AC pipe locations. This initial designation was accomplished by visually inspecting water pipe maps in the immediate vicinity of the birth residence. “Unexposed” individuals were selected from births to women living in unaffected homes and were frequency matched on the month and year of birth to exposed individuals. This process provided a tentative exposure designation until more extensive assessments (described below) were completed.

Recruitment letters were mailed to successfully located individuals along with questionnaires to gather information on demographic characteristics, sources of solvent exposure, medical histories, behavioral factors, and a detailed residential history. Additional information including the subject’s date of birth, parents’ demographic characteristics, and maternal solvent exposure was obtained from birth records and questionnaires completed by mothers in 2002–2003 for a study of developmental outcomes in this population.

Among participants available for testing from the initial cohort, only singletons currently residing within the testing area with maternal questionnaire data were eligible for the vision examinations (Table 1). Individuals who reported severe hearing or vision problems, excessive drug or alcohol use, history of neurological disease, or occupational or environmental exposure to solvents were ineligible (Table 1). Of 112 exposed participants and 107 unexposed participants who met the eligibility criteria, 56% and 61%, respectively, never responded to any of our contact attempts (i.e., three letters and several telephone calls). Another 15% of exposed participants and 8% of unexposed participants refused to participate. Ultimately, 65 participants underwent vision testing.

**Table 1:** Selection and enrollment of study population (*n*).

Exposed^a^	Unexposed^a^	Total
Selected for testing		619		626		1,245
Excluded from testing						
Outside geographic area		167		179		346
No maternal questionnaire data		126		157		283
Only postnatal exposure		44		34		78
Multiple birth		7		16		23
Otherb		153		132		285
Eligible for testing		112		107		219
Unable to contact		63		65		128
Refused		17		9		26
Underwent testing		32		33		65
Exclusion from analysis		7		4		11
Maternal occupational exposure		7		3		10
Poor acuity		0		1		1
Final analytic sample						
Final exposure statusc						
Exposed during pre- and postnatal period		25		4		29
Unexposed		0		25		25
aBased on initial exposure assessment. bReported use of ≥ 2 illicit drugs, n = 168; excessive alcohol use (e.g., average daily volume > 3 drinks), n = 162; history of neurological disease, n = 123; possible occupational exposure to solvents, n = 121; severe hearing or vision problems, n = 20; other environmental exposure to solvents, n = 14; other, n = 4. Exclusions do not add up to the total excluded because some participants are counted in multiple categories. cBased on questionnaire data and in-depth exposure assessment.						

*PCE exposure assessment.* A leaching and transport algorithm developed by Webler and Brown (1993) for our prior epidemiological studies (e.g., Aschengrau et al. 2008) was used to estimate the amount of PCE delivered to each reported residence during the subject’s gestation and through five years of age.

The components of the algorithm included the initial amount of PCE in the liner, the age of the pipe, the leaching rate of PCE from the liner into the water, and estimates of the direction and rate of flow of water derived from EPANET, water distribution modeling software developed by the U.S. EPA (Rossman 1994), which accounts for the pipe configuration and number of users in a water system. The initial amount of PCE in the liner was determined based on the pipe’s diameter and length. Laboratory experiments suggested that the leaching rate of PCE from the vinyl liner into the water declined as the pipe aged. The leaching rate followed a simple first-order exponential decay relationship with a diffusion rate constant of 2.25 years and a half-life of 1.56 years (Demond 1982; Gallagher et al. 2011). Because the study area was predominantly residential, we assumed that each residence on the distribution network used the same amount of water.

Using these data we estimated the mass of PCE delivered to subject residences for each year of the study period, based on residential move-in and pipe installation years. We calculated cumulative exposure during gestation and early childhood as the sum of nine-twelfths of the estimated mass of PCE delivered to the residence during the birth year (representing an average 9-month gestation) and the estimated mass of PCE from the month and year following birth to the month and year of the fifth birthday. Exposure assessments beyond the fifth birthday could not be conducted with confidence for participants born at the end of the study period because of limitations in available water system records. We used simple percentages to account for partial years. Individuals with no PCE exposure using the algorithm were considered unexposed. Following this exposure assessment, four individuals initially thought to be unexposed were reclassified as exposed (Table 1).

We classified exposed individuals into “low” and “high” exposure groups using a cut point of 78.4 g that corresponded to being exposed to an average drinking-water PCE concentration of 40 µg/L with an average household use of 90,000 gallons/year during gestation and early childhood. Because few families moved from exposed to unexposed residences immediately following the birth of the subject, all participants with prenatal exposure also had some childhood exposure. Thus, we were unable to examine the independent impact of prenatal exposure alone.

*Vision tests.* Separate tests were administered to assess acuity, contrast sensitivity, and color discrimination by a trained examiner who was blinded to the exposure status of each subject. During the vision examinations, participants were required to wear their best corrective lenses for near viewing. Because acquired visual dysfunction can be unilateral, all tests were administered separately for each eye, except for the Farnsworth D-15 (Bowman 1982), which was administered binocularly. All tests were conducted under standardized conditions consisting of an examination room illuminated by a daylight fluorescent lamp providing luminescence of 70 foot-Lamberts (corrected color temperature of 6,500° K; color rendering index > 90; intensity = 1,150 lux).

Near acuity test. The Rosenbaum Pocket Vision Screener (Grass Instruments Co., Quincy, MA) was used to assess acuity. A perfect acuity score is considered 20:20, and higher scores indicate poorer acuity. Participants placed their chin on the head support of the test card holder that held the card 14 inches from their eyes. With their left eye covered with a handheld occluder, participants read each number on the card progressing from top left to bottom right, beginning with the third row. Acuity score was obtained from the last row for which all numbers were correctly identified. The test was repeated for the right eye. Acuity scores for each eye were converted to LogMAR units.

Near contrast sensitivity test. The Functional Acuity Contrast Test (FACT; Stereo Optical Co., Chicago, IL) was used to assess contrast sensitivity. The FACT examination chart consists of five rows of eight sine-wave grating patches arranged in order of decreasing contrast in 0.15 log unit steps. Patches are arranged in order of increasing spatial frequency from low frequency [1.5 cycles per degree (cpd)] to intermediate frequencies (3 and 6 cpd) to high frequencies (12 and 18 cpd). Grating bars on each patch are either vertical or tilted 15 degrees to the left or right. The calibrated holder was used to hold the FACT chart 18 inches from the participants’ eyes. With their right eye covered with the occluder, for each row of grating patches participants were asked to state the orientation of the bars for the patch furthest to the right that they could see clearly. If they were correct, they continued to the right stating the orientation of grating bars for each patch in the row. If they were incorrect, they were asked to look back at each preceding patch until they gave a correct response. The raw contrast score was translated from the last correctly identified grating orientation at each spatial frequency. The test was repeated for the left eye.

Color discrimination. Both the Farnsworth D-15 and Lanthony Desaturated 15 Hue (D-15d) tests (Geller 2001) were administered to participants to assess color discrimination according to recommended protocols. For both tests, participants were shown a rectangular box containing 16 colored magnetic caps arranged in chromatic order. Participants practiced manipulating the caps with the magnetic wand before the test. The examiner then scrambled the caps in front of the subject and correctly positioned the first cap. Participants were then asked to order the remaining caps in a regular color series. They were permitted to reorder caps at any time. When they finished, the box was flipped, and the cap order was documented. Numbers of transpositions of adjacent caps (minor errors) and cap reversals across two or more cap positions (major errors) were recorded. Because the distance in color and perceived space between each successive cap is not equivalent, not all errors represent the same level of deficiency in color discrimination. Bowman (1982) and Geller (2001) published estimates of the perceptual distances between each pair of caps for the Farnsworth and Lanthony tests, respectively. A Total Color Distance Score (TCDS) was calculated as the sum of the published perceptual distances between each pair of caps in the order placed by participants. A Color Confusion Index (CCI) was calculated for each subject as the ratio of their TCDS to the TCDS associated with a perfect performance (116.9 and 56.4 for the Farnsworth and Lanthony tests, respectively) such that a CCI of 1.0 indicates a perfect score for either test. The Farnsworth test, which consists of color caps that are more vivid than those of the Lanthony test, was completed binocularly first. Then the Lanthony test was completed separately for each eye. Participants chose which eye to test first. An adhesive patch covered the other eye during this test.

*Statistical analysis.* To avoid confounding by excessive optical blur, individuals with acuity scores worse than 20:70 in either eye were excluded from analyses (*n* = 1) (Table 1). To focus our assessment on exposure to PCE-contaminated drinking water, we also excluded individuals whose mother reported occupational exposure to solvents before or during their gestation (*n* = 10). Descriptive statistics were calculated for remaining participants.

The unit of analysis for each test was the average score of the left and right eyes, except for the Farnsworth test where raw CCI scores were analyzed. Linear regression models were used to estimate the mean differences [95% confidence intervals (CIs)] in acuity, contrast sensitivity at each spatial frequency, and CCI between exposed and unexposed participants. Differences in contrast sensitivity and CCI between the high- and low-exposure groups were also calculated. We also performed a repeated-measures profile analysis with an interaction term between PCE exposure group and spatial frequency assuming unstructured covariance to determine whether exposed and unexposed participants had different patterns of contrast sensitivity across the five spatial frequencies.

Confounders were identified as Table 2 characteristics that met each of the following conditions: They preceded the exposure window, differed by > 10% between exposed and unexposed participants, and was an independent predictor of the outcome of interest (< 0.05), and inclusion in a multivariate model resulted in a > 10% change in the effect estimate for PCE exposure. No confounders were identified in our analyses of acuity or color confusion, so crude results are presented. Sex was identified as a possible confounder of the relationship between PCE exposure and contrast sensitivity, but only at spatial frequency 1.5 cpd. Therefore, crude contrast sensitivities are presented.

**Table 2:** Study population characteristics by exposure status.

Exposed		Low (n = 14)		High (n = 15)		Unexposed	
Characteristic	n (% or mean ± SD)		n (% or mean ± SD)		n (% or mean ± SD)		n (% or mean ± SD)	
Age when tested (years)		29	(30.6 ± 3.5)		14	(30.3 ± 3.2)		15	(30.9 ± 3.9)		25	(30.0 ± 3.4)
Body mass index (kg/m2)		29	(26.9 ± 5.3)		14	(28.8 ± 6.3)		15	(25.0 ± 3.2)		25	(25.7 ± 5.1)
Sex												
Female		20	(69.0)		11	(78.6)		9	(60.0)		19	(76.0)
Male		9	(31.0)		3	(21.4)		6	(40.0)		6	(24.0)
Race												
White		29	(100.0)		14	(100.0)		15	(100.0)		25	(100.0)
Education												
High school graduate or less		1	(3.4)		0	(0.0)		1	(6.7)		4	(16.0)
Some college		4	(13.8)		3	(21.4)		1	(6.7)		3	(12.0)
≥ 4 years of college		24	(82.8)		11	(78.6)		13	(86.7)		18	(72.0)
History of hypertension												
Yes		3	(10.3)		2	(14.3)		1	(6.7)		3	(12.0)
No		26	(89.7)		12	(85.7)		14	(93.3)		22	(88.0)
Solvent-exposed hobby												
Ever		27	(93.1)		13	(92.9)		14	(93.3)		23	(92.0)
Never		2	(6.8)		1	(7.1)		1	(6.7)		2	(8.0)
Alcohol frequency in previous 30 days												
1–8 days		14	(50.0)		8	(57.1)		6	(42.9)		15	(60.0)
> 8 days		9	(32.1)		2	(14.3)		7	(50.0)		7	(28.0)
None		5	(17.9)		4	(28.6)		1	(7.1)		3	(12.0)
Missing		1			0			1			0	
Smoked regularlya												
Ever		6	(20.7)		1	(7.1)		5	(33.3)		7	(28.0)
Never		23	(79.3)		13	(92.9)		10	(66.7)		18	(72.0)
Marijuana use												
Ever		13	(50.0)		4	(33.3)		9	(64.3)		19	(79.2)
Never		13	(50.0)		8	(66.7)		5	(35.7)		5	(20.8)
Missing		3			2			1			1	
Major drugsb												
Ever		4	(13.8)		0	(0.0)		4	(26.7)		4	(16.0)
Never		25	(86.2)		14	(100.0)		11	(73.3)		21	(84.0)
Maternal age at birth of subject (years)c		29	(29.0 ± 5.0)		14	(28.5 ± 4.6)		15	(29.5 ± 3.9)		25	(27.2 ± 4.2)
Paternal age at birth of subject (years)c		29	(31.8 ± 6.3)		14	(30.3 ± 4.3)		15	(33.3 ± 7.6)		25	(30.1 ± 6.1)
Maternal educational levelc												
High school graduate or less		4	(13.8)		1	(7.1)		3	(20.0)		5	(20.8)
Some college		12	(41.4)		7	(50.0)		5	(33.3)		9	(37.5)
≥ 4 years of college		13	(44.8)		6	(42.9)		7	(46.7)		10	(41.7)
Missing		0			0			0			1	
Paternal occupationc												
White collar		19	(65.5)		7	(50.0)		12	(80.0)		12	(48.0)
Blue collar		7	(24.1)		5	(35.7)		2	(13.3)		7	(28.0)
Other		3	(10.3)		2	(14.3)		1	(6.7)		6	(24.0)
aEver regular smokers were identified from affirmative answers to questions asking about smoking on “a regular basis.” bEver major drug use was defined as any use as a teen or adult of at least one of the following: inhalants, heroin, crack/cocaine, psychedelics/hallucinogens, Ritalin without a prescription, and club drugs/designer drugs (e.g., Special K, Ecstacy). cInformation on parental age, maternal education, and paternal occupation were obtained from birth records or questionnaires completed by participants’ mothers.												

Effect measure modification by smoking status was assessed by stratification. Participants were classified as ever regular smokers or never regular smokers based on their answers to specific questions about smoking on “a regular basis.” All analyses were performed using PC-SAS (version 9.1; SAS Institute Inc., Cary, NC). An alpha level of 0.05 was used as the cut point for statistical significance.

## Results

Distributions of characteristics by exposure status are presented in Table 2. Overall, the study population was white (100%), young (mean age = 30.4 ± 3.4 years), and well educated, with ≥ 4 years of college (78%). None of the participants had subjective visual complaints or a diagnosis of cataracts, macular degeneration, diabetic retinopathy, or congenital color blindness. Overall, 54% of participants (*n* = 29) were exposed to PCE-contaminated drinking water during gestation and early childhood, with 52% (*n* = 15) in the high-exposure group and 48% (*n* = 14) in the low-exposure group. Compared with unexposed participants, PCE-exposed participants were more likely to be male, not to have smoked marijuana, and to have a father with a white-collar occupation.

*Visual acuity*. The majority of both exposed (90%) and unexposed (92%) participants had 20:20 vision [logMAR (logarithm of the minimum angle of resolution) = 0]. There were no meaningful differences in visual acuity between exposed (0.03 ± 0.06 logMAR) and unexposed (0.02 ± 0.07 logMAR) participants (*p* = 0.69).

*Contrast sensitivity*. Mean contrast sensitivities for exposed and unexposed participants by spatial frequency are shown in Table 3. Although mean contrast sensitivities were lower (poorer) for PCE-exposed participants than unexposed participants at intermediate and high spatial frequencies, differences were not statistically significant, except at the highest frequency of 18 cpd. We obtained similar results when the repeated-measures analysis took into account the correlation between spatial frequencies. Although there was some variation in patterns across spatial frequency by exposure group, there was no statistically significant interaction (*p* = 0.08) (data not shown).

**Table 3:** Mean (± SD) contrast sensitivities by spatial frequency for PCE-exposed and unexposed participants.

Spatial frequency (cpd)	Exposed (n = 29)	Unexposed (n = 25)	Mean difference (95% CI)	p-Value
1.5		60.88 ± 18.47		55.34 ± 11.96		5.54 (–3.12, 14.19)		0.20
3.0		101.72 ± 25.28		107.74 ± 24.69		–6.02 (–19.71, 7.68)		0.38
6.0		107.47 ± 31.02		112.84 ± 29.84		–5.37 (–22.07, 11.32)		0.52
12.0		42.98 ± 18.94		50.88 ± 24.04		–7.90 (–19.6, 3.84)		0.18
18.0		15.30 ±7.17		21.78 ± 13.45		–6.47 (–12.33, –0.62)		0.03

Comparisons by PCE exposure level revealed that the decreased contrast sensitivity was generally restricted to the high exposure group with little difference between the low exposure and the unexposed groups (Table 4). Following stratification by regular smoking status, we found evidence of poorer mean contrast sensitivities for PCE-exposed compared with unexposed participants across most spatial frequencies (3, 6, 12, and 18 cpd) among never regular smokers (*n* = 41) [see Supplemental Material, Table S1 (http://dx.doi.org/10.1289/ehp.1103996)], though differences were not statistically significant. No consistent pattern was observed among ever regular smokers (*n* = 13); however, effect estimates were very imprecise because of the small number of participants in this smoking category.

**Table 4:** Mean (± SD) contrast sensitivities and mean differences (95% CIs) by spatial frequency according to exposure level.

Spatial frequency (cpd)	High (n = 15)	Low (n = 14)	Unexposed (n = 25)	High vs. unexposed mean difference (95% CI)	Low vs. unexposed mean difference (95% CI)	High vs. low mean difference (95% CI)
1.5		60.23 ± 18.86		61.57 ± 18.73		55.34 ± 11.96		4.89 (–5.56, 15.35)		6.23 (–4.46, 16.92)		–1.34 (–13.24, 10.56)
3.0		94.13 ± 23.65		109.86 ± 25.23		107.74 ± 24.69		–15.72 (–34.04, 2.59)		2.11 (–14.33, 18.57)		–13.61 (–29.70, 2.49)
6.0		102.33 ± 32.84		112.96 ± 29.12		112.84 ± 29.84		–10.51 (–30.52, 9.50)		0.12 (–20.33, 20.57)		–10.63 (–33.40, 12.14)
12.0		38.20 ± 17.41		48.11 ± 19.78		50.88 ± 24.04		–12.68 (–26.66, 1.30)		–2.77 (–17.06, 11.52)		–9.91 (–25.82, 6.00)
18.0		14.71 ± 7.37		15.89 ± 7.18		21.78 ± 13.45		–7.07 (–14.23, 0.10)		–5.89 (–13.06, 1.28)		–1.18 (–9.30, 6.94)

*Color discrimination*. Farnsworth D-15 test. Most of the exposed (76%) and unexposed (92%) participants achieved perfect scores on the Farnsworth test. Only 3 participants, all of whom were exposed to PCE, made major positioning errors. The mean difference in Farnsworth CCI between PCE-exposed and -unexposed participants was 0.05 (95% CI: 0.003, 0.10; *p* = 0.04), indicating poorer color discrimination among exposed participants (Table 5). Comparisons by PCE exposure level revealed reduced color discrimination for both the high- and low-exposure groups compared with the unexposed group, but the difference in Farnsworth CCI was larger and statistically significant for the high-exposure group (Table 6).

**Table 5:** Mean color confusion index for PCE-exposed and unexposed participants.

Test	Exposed (n = 29) mean ± SD	Unexposed (n = 25) mean ± SD	Mean difference (95% CI)	p-Value
Farnsworth		1.05 ± 0.12		1.00 ± 0.02		0.05 (0.003, 0.10)		0.04
Lanthony		1.16 ± 0.19		1.10 ± 0.09		0.07 (–0.02, 0.15)		0.11

**Table 6:** Mean color confusion index and mean differences (95% CIs) according to PCE exposure level.

Test	High (n = 15) mean ± SD	Low (n = 14) mean ± SD	Unexposed (n = 25) mean ± SD	High vs. unexposed mean difference (95% CI)	Low vs. unexposed mean difference (95% CI)	High vs. low mean difference (95% CI)
Farnsworth		1.07 ± 0.13		1.04 ± 0.10		1.00 ± 0.02		0.06 (0.006, 0.120)		0.04 (–0.022, 0.094)		0.03 (–0.037, 0.092)
Lanthony		1.20 ± 0.20		1.12 ± 0.18		1.10 ± 0.09		0.10 (0.005, 0.204)		0.03 (–0.073, 0.131)		0.08 (–0.038, 0.189)

In analyses stratified by regular smoking status, the mean Farnsworth CCI estimates were higher for PCE-exposed than unexposed participants among both ever (*n* = 13) and never regular smokers (*n* = 41), but a larger difference was observed among ever regular smokers [see Supplemental Material, Table S2 (http://dx.doi.org/10.1289/ehp.1103996)].

Lanthony D-15d test. Minor errors in cap positioning on the Lanthony test were made by most exposed (79%) and unexposed participants (76%). Approximately 41% of PCE-exposed participants made major errors compared with only 28% of unexposed participants. All major errors were consistent with deficits in blue–yellow color discrimination. Mean CCI was poorer among PCE-exposed than unexposed participants (mean difference = 0.07; 95% CI: –0.02, 0.15), but the difference was not statistically significant (Table 5). As seen for the Farnsworth test, comparisons by PCE exposure level revealed that differences in Lanthony CCI were greater for the high-exposure group than the low-exposure group, but difference between the two exposure levels was not statistically significant (Table 6).

Stratification by smoking status revealed that mean CCI estimates were higher for PCE-exposed than unexposed participants among both ever (*n* = 13) and never smokers (*n* = 41), but a larger, statistically significant difference between PCE-exposed versus PCE-unexposed participants was observed among ever regular smokers (mean difference = 0.25; 95% CI: 0.04, 0.47) [see Supplemental Material, Table S2 (http://dx.doi.org/10.1289/ehp.1103996)].

## Discussion

Our results suggest that exposure to PCE-contaminated drinking water during gestation and early childhood may be associated with long-term subclinical visual decrements in adulthood. PCE-exposed participants had reduced contrast sensitivity and poorer color discrimination compared with unexposed participants, although differences between exposed and unexposed participants were generally not statistically significant.

The reduced contrast sensitivity in intermediate spatial frequencies observed in this population is consistent with findings among infants and young children born to women exposed occupationally to solvent mixtures during pregnancy (Till et al. 2005), PCE-exposed apartment dwellers and day-care workers (Schreiber et al. 2002), and solvent-exposed workers (Donoghue et al. 1995; Frenette et al. 1991; Gong et al. 2003; Mergler et al. 1991). In the current study, reductions in contrast sensitivity at intermediate spatial frequencies were restricted to the highest exposure group, whereas others have observed reductions among children with prenatal exposure to be independent of exposure level. The reason for this discrepancy is unclear, but may be attributable to differences in the specific solvent being assessed or to the exposure level cut points.

With respect to color vision, the decrements we observed among adults exposed prenatally and in early life agree with observations among adults exposed occupationally (Gobba et al. 1998; Sharanjeet et al. 2004). We found a larger difference in CCI among the high PCE–exposure group than the low-exposure group when each was compared with the unexposed group. This finding supports a possible dose effect. Results from previous studies that assessed a dose–response relationship have been inconsistent. A prior study found no evidence of a relationship between reduced color discrimination among children and maternal exposure intensity during pregnancy, derived as a weighted score based on factors including type of solvent, proximity to exposure, and duration of exposure (Till et al. 2001). However, in a study among children residing in buildings shared by dry cleaner establishments, PCE levels in breath samples correlated with CCI and frequency of major errors on the Lanthony test [New York State Department of Health (NYSDOH) 2005].

Others have found that prenatal exposure to mixed solvents is associated with poorer red–green color discrimination among infants (Till et al. 2005) and poorer red–green and blue–yellow discrimination among young children (Till et al. 2001). Our present results are concordant with the latter, because only exposed participants made major errors on the Farnsworth test, considerably more exposed than unexposed participants made major errors on the Lanthony test, and all major positioning errors on either test were on the blue–yellow axis. Specific major errors made on the Lanthony test by exposed and unexposed participants in our study are similar to those observed among a cohort of microelectronic workers (Geller and Hudnell 1997). Studies assessing whether color vision changes associated with adult occupational solvent exposure are reversible have found conflicting results (Cavalleri and Gobba 1998; Gobba 2000). A follow-up evaluation among children who attended a day care in a building shared with a dry cleaner found that contrast sensitivity and color vision were normal and similar to that of unexposed children about 4.5 years after exposure to air concentrations of 1,800–2,400 µg/m^3^ PCE had stopped (NYSDOH 2005). In contrast, our findings among adults suggest that the effects of early-life PCE exposure on color discrimination may be irreversible.

Our results should be considered in light of limitations. Cumulative exposure estimates depended on the accuracy of variables in the leaching and transport algorithm and water modeling software. Because exposure was calculated similarly for all participants before assessments of visual function, any exposure misclassification was likely nondifferential with regard to the outcome. In fact, questionnaire responses indicated that most participants and their parents did not know whether their drinking-water supply was contaminated. The contribution of other sources of solvent exposure after 5 years of age (e.g., jobs and hobbies) was considered in the analysis.

Although results from our validation studies indicate reasonable correlation between our exposure estimates and PCE concentrations in historical water samples (Gallagher et al. 2011; Spence et al. 2008), nondifferential exposure misclassification would bias dichotomous comparisons towards no effect. Nondifferential misclassification would result in an unpredictable bias for associations with low PCE exposure, but would underestimate associations with high PCE exposure (Rothman et al. 2008). Although our exposure measure did not account for differences in water consumption and bathing habits because recall of these characteristics was poor, not adjusting for such factors was unlikely to have appreciably influenced exposure ranking (Vieira et al. 2005). Participation rates were much lower than expected, which would have hindered our ability to detect subclinical differences in vision. However, nonparticipants were similar to participants with respect to exposure status (63% vs. 54% exposed), maternal age at birth (mean age = 28.5 years vs. 28.2 years), sex (67% vs. 72.2% female), race (99% vs. 100% white), education (70% vs. 78% with ≥ 4 years of college), and frequent use of self-service dry cleaning (19.0% vs. 19.2%). Subanalyses stratified by smoking status resulted in strata with small numbers leading to imprecise effect estimates for PCE exposure, especially for ever regular smokers.

Although we collected information on many potential confounders, there was little evidence of actual confounding by the factors evaluated. Observation bias was unlikely because the examiner was masked to the subject’s exposure status. Most studies assessing the effects of solvent exposure on vision have focused on exposure via inhalation, whereas our analysis examined exposure mainly from ingestion of contaminated water and, to a lesser extent, dermal absorption and inhalation during bathing. Thus, it is possible that our findings are not generalizable to populations exposed in early life by other routes.

## Conclusions

PCE has been implicated in deficiencies of contrast sensitivity and color discrimination among adults with occupational and environmental exposure and children with prenatal exposure. To the best of our knowledge, this is the first study to assess the associations between prenatal and early-childhood exposure to PCE and adult vision. Our results suggest that exposure to PCE via drinking water during these critical periods of development may be associated with long-term subclinical visual dysfunction in adulthood, particularly color discrimination. Our study has limitations, particularly a small sample size and possible exposure misclassification, so further investigation of similarly exposed populations is necessary to substantiate these findings.

## Supplemental Material

(61 KB) PDF
