# Supplementary material for: Prenatal and Early Childhood Exposure to Tetrachloroethylene and Adult Vision
Source: Environ Health Perspect. 2012 Jul 11;120(9):1327–32. doi: 10.1289/ehp.1103996 (PMC3440105; doi:10.1289/ehp.1103996)
Supplement: (61 KB) PDF [file ehp.1103996.s001.pdf]

SUPPLEMENTAL MATERIALS

Prenatal and Early Childhood Exposure to Tetrachloroethylene and Adult Vision

Kelly D Getz[1], Patricia A Janulewicz[1], Susannah Rowe[2], Janice M Weinberg[3], Michael R Winter[4], Brett R Martin[4], Veronica M Vieira[5], Roberta F. White[5,6], Ann Aschengrau[1]

[1] Department of Epidemiology Boston University School of Public Health, Boston MA 02118
[2] Department of Ophthalmology, Boston University School of Medicine, Boston, MA 02118
[3] Department of Biostatistics, Boston University School of Public Health, Boston, MA 02118
[4] Data Coordinating Centers, Boston University School of Public Health, Boston, MA 02118
[5] Department of Environmental Health, Boston University School of Public Health, Boston MA 02118
[6]Department of Neurology, Boston University School of Medicine, Boston, MA 02118

TABLE OF CONTENTS

Supplemental Material, Table S1. Mean Contrast Sensitivity at Each Spatial Frequency for PCE-exposed and Unexposed Subjects Stratified by Smoking Status………… 2

Supplemental Material, Table S2. Mean Color Confusion Index for PCE-exposed and Unexposed Subjects Stratified by Smoking Status………… 3

Supplemental Material, Table S1. Mean Contrast Sensitivity at Each Spatial Frequency for PCE-exposed and Unexposed Subjects Stratified by Smoking Status

	Ever Regular Smoker (N=13)			Never Regular Smoker (N=41)		
	Exposed (n=6)	Unexposed (n=7)		Exposed (n=23)	Unexposed (n=18)	
Spatial Freq (cpd)	Mean +/-SD	Mean +/-SD	Difference (95% CI)	Mean +/-SD	Mean +/-SD	Difference (95% CI)
1.5	65.8 +/-16.2	58.6 +/-10.9	7.1 (-9.5, 23.7)	59.6 +/-19.1	54.1 +/-12.4	5.6 (-5.0, 16.2)
3	100.8 +/-27.7	99.4 +/-25.9	1.3 (-31.4, 34.0)	102.0 +/-25.3	111.0 +/-24.2	-9.0 (-24.8, 6.8)
6	97.5 +/-41.0	99.0 +/-38.8	-1.5 (-50.3, 47.3)	110.1 +/-28.4	118.2 +/-24.8	-8.2 (-25.3, 9.0)
12	44.0 +/-24.4	40.5 +/-27.8	3.5 (-28.7, 35.7)	42.7 +/-17.9	54.9 +/-22.0	-12.2 (-24.8, 0.4)
18	15.4 +/-5.6	24.5 +/-19.5	-9.1 (-29.4, 11.2)	15.3 +/-7.6	20.7 +/-10.8	-5.4 (-11.2, 0.4)

Supplemental Material, Table S2. Mean Color Confusion Index for PCE-exposed and Unexposed Subjects Stratified by Smoking Status

	Ever Regular Smoker (N=13)			Never Regular Smoker (N=41)		
	Exposed (n=6)	Unexposed (n=7)		Exposed (n=23)	Unexposed (n=18)	
Test	Mean +/-SD	Mean +/-SD	Mean Difference (95% CI)	Mean +/-SD	Mean +/-SD	Mean Difference (95% CI)
Farnsworth	1.12 +/-0.16	1.01 +/-0.03	0.11 (-0.02, 0.25)	1.03 +/-0.10	1.00 +/-0.01	0.03 (-0.01, 0.08)
Lanthony	1.34 +/-0.23	1.09 +/-0.12	0.25 (0.04, 0.47)	1.12 +/-1.12	1.10 +/-0.08	0.02 (-0.06, 0.10)
